# Assessment of oral health-related quality of life and oral side effects of radioactive iodine therapy

**DOI:** 10.1007/s00784-025-06201-y

**Published:** 2025-02-08

**Authors:** Hatice Yemenoglu, Kadriye Peker, Taha Emre Köse, Dilara Nil Günaçar, Ogün Bülbül

**Affiliations:** 1 Department of Periodontology, Faculty of Dentistry, Recep Tayyip Erdoğan University, Islampasa Street, Menderes Boulevard, Rize, 53020 Turkey; 2 Department of Basic Medical Science, Faculty of Dentistry, Istanbul University, Istanbul, Turkey; 3 Department of Oral and Maxillofacial Radiology, Faculty of Dentistry, Recep Tayyip Erdoğan University, Rize, Turkey; 4 Department of Nuclear Medicine, Faculty of Medicine, Recep Tayyip Erdoğan University, Training and Research Hospital, Rize, Turkey

**Keywords:** Dental caries; oral health-related quality of life, Periodontal diseases, Radioactive iodine treatment, Thyroid neoplasms; xerostomia

## Abstract

**Objectives:**

To evaluate the impact of oral health problems on oral health-related quality of life (OHRQoL) among female patients received Radioactive iodine (^131^I) therapy.

**Materials and methods:**

This unmatched case-control study was conducted on 40 female patients (20 cancer free controls and 20 patients treated with ^131^I therapy). Data were collected via clinical examination, self reported questionnaire including the Oral Health Impact Profile-14 (OHIP-14), salivary tests, socio-demographic and behavioural characteristics. Data were analyzed using descriptive, bivariate and multivariate statistics.

**Results:**

There were significant differences in the total number of decayed, missing and filled surfaces, stimulated and unstimulated salivary flow rates, and periodontal indices between the study and control groups in the unadjusted analysis. Age adjusted analysis revealed significant differences in the stimulated and unstimulated salivary flow rates, periodontal indices, physical pain domain scores between groups. No significant differences were observed between groups in the xerostomia severity and OHRQoL. In study group, the score for the OHIP-14 psychological discomfort domain was negatively correlated with both stimulated and unstimulated salivary flow rates. The total OHIP-14 score and its domain scores of physical pain and psychological disability were correlated positively with the severity of xerostomia, but negatively correlated with number of the repeated ^131^I therapy.

**Conclusions:**

Due to xerostomia, patients reported worse OHRQoL in the domains of physical pain, psychological discomfort and disability. They had worse periodontal status and tooth brushing habits than healthy controls.

**Clinical relevance:**

The findings of this study may provide a valuable insight on the oral health problems and needs of target group when planning a a team-based care.

## Introduction

Radioactive iodine (^131^I) therapy has been used successfully for years in the treatment of thyroid cancer [1]. Although ^131^I is safe and well tolerated, patients may experience potential short-term and long-term health effects, which affect patients’ longevity and quality of life (QoL) [2].

Previous studies showed that high-dose ^131^I therapy can have acute (sialadenitis, hyposalivation, salivary and lacrimal gland dysfunction, dysphagia, reducing of calcium/phosphate concentration, alteration of enamel and dentin, taste loss, and oral candidiasis) and long–term adverse effects (chronic sialadenit, permanent taste loss, xerostomia, and increased salivary gland malignancies) on patients’ oral health depending on the intensity of treatment and total uptake of radioiodine in the salivary glands [3–6]. Due to postradioiodine xerostomia, patients face many oral health problems and complications such as increasing caries risk, tooth extraction rates, eating difficulties, resources for dental foci which affected their QoL and oral cavity function. In addition, ^131^I reduces the levels of prostaglandin, which is responsible for salivation, and causes plaque formation, gingivitis and periodontal disease [3, 5, 6]. Recent evidences reported that there is a bidirectional association between thyroid diseases and periodontal disease [7, 8] and the chanching microbiota and inflammatory cytokines may increase pro-inflammatory environment among differentiated thyroid carcinoma patients with xerostomia after ^131^I therapy [8, 9].

Team-based care for cancer survivors is needed for monitoring cancer recurrence and managing long-term effects of cancer treatment [10], because many factors including the fear of cancer recurrence, long-term effects of cancer treatment, health inequalities, financial problems, the informational, emotional and psychological needs, unmeet health care needs adversely affects the health related QoL in thyroid cancer survivors [4, 6, 10–17].

Using the generic and Thyroid Cancer–Specific QoL measures, the assessment of patient-centered outcome in tyroid cancer patients and survivors is crucial for estimating the patient’s health needs, treatment related outcomes, and the effectiveness of existing health care programs or interventions as well as planning high-quality survivorship care [10, 18, 19]. This assessment may provide opportunities for oral health professionals to increase their awareness about patients’ oral health needs and oral health problems caused by iodine treatment, to develop the oral health preventive programs aimed to enhance patients’ motivation of oral hygiene and to prevent possible complications. To our knowledge, there have been no studies evaluating the long term adverse effects of ^131^I therapy on oral health status and oral health related quality of life (OHRQoL) of patients with thyroid cancer. This study aims to determine salivary flow rate, oral health status, and OHRQoL of patients receiving ^131^I therapy, define the factors affecting their OHRQoL and compare their OHRQoL to cancer free control patients. The null hypothesis assumed no significant differences in salivary paramaters, oral health status and OHRQoL between patients receiving ^131^I therapy and cancer free control patients in long term period.

## Materials and methods

This study was approved by the Ethics Committee of Recep Tayyip Erdoğan University (2020/16). The study was conducted in accordance with the 1964 Helsinki Declaration. The purpose and content of the research were explained to the individuals included in the study, and voluntary consent forms were signed.

### Patient selection

A total of 40 female participants, including 20 cancer free control patients (control group) and 20 thyroid cancer patients with a history of ^131^I therapy for well differentiated types of thyroid neoplasms (papillary thyroid carcinoma or follicular thyroid carcinoma) (study group) who applied for treatment to our faculty were included in the study. The sample size was 20 participants in each group with a power of 0.80 and a 2-tailed alpha of 0.05 for detecting a difference in the xerostomia proportions of 0.39 between control and study group [3, 20] using the online calculator [21].

Inclusion criteria for study group included being female aged between 18 and 65 years, having ^131^I therapy for different thyroid neoplasms, voluntary study participation, not having received any periodontal treatment in the last 6 months, and not having a history of different areas of cancer treatment. Controls were selected from dental patients who visited the dental hospital of the same faculty using the same selection criteria as the cases except for the having ^131^I therapy for different thyroid neoplasms. Exclusion criteria for study and control groups were as follows: having any chronic systemic disease, being a pregnant or lactating women, refusing to participate in the study, the inability to read, understand and complete the questionnaire due to illiteracy, physical and cognitive impairment. In addition, control patients who use the medicine causing xerostomia were excluded.

### Data collection

Data were collected by a trained clinician via clinical examination, salivary tests, medical record, and self reported questionnaire.

### Self-reported data

To assess the OHRQoL of patients, the Turkish version of the Oral Health Impact Profile-14 (OHIP-14), validated by Mumcu et al. for Turkish population, was used [22]. This questionnaire consist of 14 items within 7 subscales (functional limitation, physical pain, psychological discomfort, physical disability, psychological disability, social disability, and handicap), scored on 5-point Likert scale (from “0-never” to “4-very often”). The total OHIP-14 score ranges from 0 to 56, with lower scores indicating better OHRQoL.

The self-reported questionnaire including patients’ socio-demographic characteristics (education level, age, income level, marital and employment status), subjective oral health ratings (perceived treatment need, perceived periodontal health), oral health behaviours (tooth brushing frequency, dental visiting pattern), smoking and alcohol consumption habits was collected by a researcher (HY).

### Clinical data and laboratory tests

Clinical information about the patient’s RAI dose, number of repeated RAI therapy, and duration after RAI therapy were collected by the oncologist from the medical records registered in the hospital information system and epicrisis reports.

The severity of xerostomia was evaluated subjectively using the 10 cm visual analog scale (VAS) [23]. Saliva samples were collected by the same clinician between 9 and 11 am into sterile tubes by the traditional spitting method [24, 25]. Individuals refrained from oral hygiene, eating and drinking for at least 1 h before saliva collection. Patients were seated in a quiet area of the clinic, away from oral, visual and sensory stimuli. For determining unstimulated salivary flow rates, individuals were asked to transfer the saliva that had accumulated in their mouths into the provided container without swallowing for 5 min. To determine the stimulated salivary flow rate, the mouth was first rinsed with a mouthful of water for 30 s and spit out. Individuals were asked to chew a paraffin tablet and spit the saliva produced in the first 1 min into the saliva cup. The saliva formed by chewing the paraffin tablet for the next 5 min was collected in a sterile tube [24, 25]. After saliva collection, stimulated and unstimulated flow rates were calculated as ml/min by reading the amount of saliva from the striped tube.

In both groups, clinical and radiographic examinations were performed by the same clinician (HY). Before the study, 50% of the patients (*n* = 20) were performed intra-examiner calibration exercises (PD and CAL measurements) in two different periods, 2 weeks apart. Intra-examiner calibrations were evaluated with Cohen’s κ coefficient. Dental caries severity was evaluated using the Decayed Missing Filled Surfaces (DMFS) according to WHO criteria [26]. In clinical periodontal examination, probing depth (PD) and interdental clinical attachment loss (CAL) [27], bleeding on probing (BOP) [28], gingival index (GI) [29] and plaque index (PI) [30] using Williams periodontal probe were evaluated and recorded.

### Statistical analysis

Statistical analysis was performed using the SPSS 22.0 statistical package program. The Shapiro Wilk test was used to check the normality of data. Descriptive and bivariate analyses were used to analyze the data. Non-parametric tests were used for the analysis because of the non-normality of the data. The chi-squared test was to examine the differences between categorical variables. The Mann-Whitney U-Test was used to investigate the differences between groups and Spearman’s rank correlation coefficient was used to evaluate the relationship between two continuous variables. Correlation strengths were interpreted as weak (*r* ≤ 0.49), moderate (0.50 ≤ *r* ≤ 0.74), and strong (*r* ≥ 0.75) [31].

For data analysis, the following variables were treated as dichotomous: patients’s education level (≤ 8 years vs. > 8 years [32], income level (minimum wage and less vs. above minimum wage) [32], marital status (single/divorsed vs. married), smoking status (past or current smokers vs. never-smokers) [32], alcohol consumption (yes vs. no) [32], employment status (unemployed vs. employed full-time or part-time), daily tooth brushing frequency (≥ 2 vs. < 2) [33], perceived treatment need (yes vs. no) [34], perceived periodontal health (excellent/very good/good vs. fair/ bad) [35], dental visiting pattern (regular dental check up vs. problem oriented) [36], whereas age, stimulated/unstimulated salivary flow rate, subjective assesment of xerostomia severity, ^131^I dose, number of the repeated ^131^I therapy, duration time after the ^131^I therapy, the OHIP-14 (total and domain) scores, caries severity and periodontal indices scores were treated as continuous independent variables. Age is accepted as a risk factor for oral diseases due to accumulated periodontal destruction, the number of surfaces at risk for caries increases [37]. Logistic regression analysis was performed to control age covariate. Internal consistency of the OHIP-14 was measured using Cronbach’s alpha coefficient. An acceptable Cronbach’s Alpha is considered to be above 0.70 [38]. Post-hoc power analysis was performed using the online calculator ClinCalc (available at https://clincalc.com/stats/power.aspx, accessed on 03/01/2025). The *p* value < 0.05 was considered as statistically significant.

## Result

The characteristics of the study participants are presented in Table 1. A total of 20 patients (mean age 44.50 ± 10.76) and 20 controls (mean age 34.95 ± 11.39) were included in the study. Out of the patients, 16 (80%) patients were with papillary thyroid carcinoma, while 4 (20%) were with follicular thyroid carcinoma. The mean total dose of ^131^I administered was 100.33 millicuries (minimum 30 millicuries, maximum 175 millicuries). A total of 13 (65%) patients received a single dose of ^131^I; 4 (20%) patients were given 2 doses; 2 (10%) patients 3 doses; and 1 (5%) patients 4 doses. Mean duration time after the ^131^I therapy was 8.20 ± 3.76 years (ranged from 3 to 16 years).

**Table 1:** Demographic, behavioral and subjective characteristics of the study population

	Study group (*n* = 20)		Control group (*n* = 20)		*p*-value
	*n*	%	*n*	%	
**Educational level** ^**b**^					0.004*
≤ 8 years	16	80	6	30	
>8 years	4	20	14	70	
**Employment Status** ^**b**^					< 0.001*
Unemployed	20	100	9	45	
Employed full-time or part-time	0	0.00	11	55	
**Income level** ^**b**^					0.695
Minimum wage and less	3	15	5	25	
Above minimum wage	17	85	15	75	
**Marital status** ^**b**^					0.001*
Single/Divorsed	1	5	11	55	
Married	19	95	9	45	
**Age (mean ± SD)** ^**c**^		44.50 ± 10.76		34.95 ± 11.39	0.010*
**Self perceived periodontal health** ^**a**^					0.525
Bad	12	60	10	50	
Good	8	40	10	50	
**Perceived treatment need** ^**b**^					0.663
Yes	18	90	17	85	
No	2	10	3	15	
**Dental visiting patterns** ^**b**^					0.311
Regular dental chek Problem oriented	1 19	5 95	0 20	0 100	
**Daily tooth brushing** ^**b**^					0.022*
≥2 <2	4 16	20 80	12 8	60 40	
**Smoking** ^**b**^					0.487
Past or current smokers	0	0.0	2	10	
Never-smokers	20	100	18	90	

^a^The Chi*-*squared test; ^b^Fisher’s exact test; ^c^The Mann-Whitney U-Test; SD, Standard deviation

There were significant socio-demographic differences in educational level (*p* = 0.004), age (*p* = 0.010), employment (*p* < 0.001) and marital status (*p* = 0.001) between study and control group. No significant differences were found between groups with respect to self perceived periodontal health (*p* = 0.525), perceived treatment need (*p* = 0.663), dental visiting pattern (*p* = 0.311), and smoking status (*p* = 0.487), except for toothbrushing frequency (*p* = 0.022).

There was statistically significant difference in DMFS (*p* = 0.006), stimulated (*p* < 0.001), and unstimulated salivary flow rates (*p* < 0.001), PI (*p* = 0.024), GI (*p* = 0.012), BOP (*p* = 0.003), PD (*p* = 0.010) and CAL (*p* = 0.004) values between the study and control groups, except for the severity of xerostomia (VAS) (*p* = 0.134). After adjusting for age, significant differences were found in stimulated (*p* = 0.007), and unstimulated salivary flow rates (*p =* 0.008), GI (*p* = 0.040), and BOP (*p* = 0.032) (Table 2).

**Table 2:** Clinical characteristics of the study population

	Group	Mean ± SD	*p*-value*	Age-adjusted *p* value**
Stimulated salivary flow rate (ml/dk)	Study (*n* = 20)	0.48 ± 0.30	< 0.001	0.007
	Control (*n* = 20)	1.10 ± 0.48		
Unstimulated salivary flow rates (ml/dk)	Study (*n* = 20)	0.33 ± 0.22	< 0.001	0.008
	Control (*n* = 20)	0.79 ± 0.39		
PI	Study (*n* = 20)	1.84 ± 0.73	0.024	0.083
	Control (*n* = 20)	1.25 ± 0.74		
GI	Study (*n* = 20)	1.76 ± 0.68	0.012	0.040
	Control (*n* = 20)	1.20 ± 0.66		
BOP	Study (*n* = 20)	66.45 ± 26.26	0.003	0.032
	Control (*n* = 20)	38.00 ± 32.01		
PD	Study (*n* = 20)	2.32 ± 0.39	0.010	0.214
	Control (*n* = 20)	1.99 ± 0.47		
CAL	Study (*n* = 20)	2.56 ± 0.48	0.004	0.150
	Control (*n* = 20)	2.10 ± 0.56		
DMFS	Study (*n* = 20)	58.10 ± 38.48	0.006	0.227
	Control (*n* = 20)	29.85 ± 30.99		
The severity of xerostomia (VAS)	Study (*n* = 20)	2.90 ± 3.01	0.134	0.346
	Control (*n* = 20)	1.70 ± 2.79		

*The Mann-Whitney U-Test; SD, Standard deviation; PI, Plaque index; GI, Gingival index; BOP, Bleeding on probing; PD, Probing depth; CAL, Interdental clinical attachment loss; DMFS, Decayed Missing Filled Teeth Surfaces Index; VAS, Visual analog scale; **pvalues were determined by logistic regression and were adjusted for age

As seen in Table 3, no statistically significant differences were observed between study and control groups in the total the OHIP-14 score and its domain scores (*p* > 0.05). We found significant differences between groups in the domain of physical pain after adjusting for age (*p* = 0.036).

**Table 3:** Differences in the total the OHIP*-*14 score and its domain scores

OHIP-14	Study group	Control group	*p*-value*	Age-adjusted *p* value**
	Mean ± SD	Mean ± SD		
Physical pain	4.20 ± 4.18	2.15 ± 2.16	0.149	0.036
Psychological discomfort	2.25 ± 2.86	1.30 ± 2.05	0.398	0.285
Functional limitation	2.10 ± 2.92	0.75 ± 1.12	0.445	0.053
Psychological disability	1.55 ± 2.35	0.65 ± 1.84	0.221	0.189
Social disability	1.55 ± 1.96	0.75 ± 1.52	0.242	0.067
Handicap	0.40 ± 0.88	0.80 ± 0.95	0.211	0.316
Total score	12.05 ± 11.94	6.40 ± 6.67	0.157	0.063

* The Mann-Whitney U-Test; SD, Standard deviation; **p values were determined by logistic regression and were adjusted for age

In study group, the score for the OHIP-14 psychological discomfort domain was negatively correlated with both stimulated (*r* = -0.575;*p* < 0.01) and unstimulated salivary flow rates (*r* = -0.593;*p* < 0.01), whereas the severity of xerostomia was positively correlated with the total OHIP-14 score (*r* = 0.473;*p* < 0.05) and its domain scores of physical pain (*r* = 0.459;*p* < 0.05) and psychological disability (*r* = 0.562;*p* < 0.01). The total OHIP-14 score (*r* = -0.498;*p* < 0.05) and its domain scores of physical pain (*r* = -0.542;*p* < 0.05) and psychological disability (*r* = -0.562; *p* < 0.01) were negatively correlated with number of the repeated RAI therapy (Table 4).

**Table 4:** Correlations between clinical paramaters and OHIP-14 in study and control groups

	Physical pain *r*	Psychological discomfort *r*	Functional limitation *r*	Psychological disability *r*	Social disability *r*	Handicap *r*	Total score *r*
**Study group**							
Age	-0.115	0.010	0.009	0.083	-0.161	0.040	-0.027
DMFS	0.363	0.121	0.404	0.047	0.128	-0.196	0.369
The severity of xerostomia (VAS)	0.459*	0.382	0.307	0.562**	0.116	0.197	0.473*
*S*timulated salivary flow rate (ml/dk)	-0.066	-0.575**	-0.043	-0.208	-0.016	-0.222	-0.202
Unstimulated salivary flow rate (ml/dk)	-0.056	-0.593**	0.038	-0.192	0.013	-0.201	-0.159
PI	-0.281	-0.336	-0.006	-0.319	-0.177	-0.126	-0.206
GI	-0.173	-0.194	0.041	-0.068	0.052	0.159	-0.002
BOP	-0.244	-0.361	-0.117	-0.425	-0.058	-0.213	-0.206
PD	-0.383	-0.066	-0.121	-0.192	-0.039	0.149	-0.185
CAL	-0.420	-0.020	-0.233	-0.189	-0.139	0.062	-0.241
RAI dose (mCi)	-0.417	0,024	-0.018	0.352	0.018	0.300	-0.073
Number of the repeated RAI therapy	-0.542*	-0.327	-0.218	-0.562**	-0.357	-0.356	-0.498*
Duration time after the RAI therapy	-0.187	0.277	-0.095	-0.022	0.170	0.288	-0.089
**Control Group**							
Age	-0.164	0.208	-0.456*	0.170	-0.281	-0.122	-0.155
DMFS	-0.054	0.304	-0.390	-0.025	-0.206	-0.129	-0.091
The severity of xerostomia (VAS)	-0.107	0.134	-0.116	0.156	0.139	0.189	0.046
*S*timulated salivary flow rate (ml/dk)	-0.116	-0.292	0.181	-0.071	0.043	0.154	-0.071
Unstimulated salivary flow rate (ml/dk)	0.128	-0.357	0.223	0.009	0.249	0.273	0.104
PI	-0.200	0.023	0.058	-0.017	-0.402	-0.309	-0.183
GI	-0.142	-0.018	0.086	-0.009	-0.381	-0.244	-0.160
BOP	-0.319	-0.099	0.028	-0.190	-0.635**	-0.378	-0.376
PD	-0.141	-0.070	-0.267	-0.014	-0.561*	-0.375	-0.325
CAL	-0.168	-0.078	-0.256	-0.006	-0.594**	-0.270	-0.293

r, Spearman’s rank correlation coefficient; DMFS, Decayed Missing Filled Teeth Surfaces Index; VAS, Visual analog scale; PI, Plaque index; GI, Gingival index; BOP, Bleeding on probing; PD, Probing depth; CAL, Interdental clinical attachment loss; mCi, Milicurie

In the control group, the findings revealed that the social disability domain of the OHIP-14 correlated negatively and significantly with BOP (*r* = -0.635;*p* < 0.01), PD (*r* = -0.561;*p* < 0.01), and CAL (*r* = -0.594; *p* < 0.01). Age was correlated negatively with the functional limitation domain score (*r* = -0.456;*p* < 0.05) (Table 4). The observed correlations between continnous variables in both two groups ranged from weak (*r* ≤ 0.49) to moderate (0.50 ≤ *r* ≤ 0.74) magnitude.

For the re-evaluation of PD and CAL measurements, the weighted κ coefficients were found to be 0.872 and 0.890, respectively, with good intra-examiner reliability [39]. In this study, the Cronbach’s alpha (α) coefficient for the OHIP-14 was found to be 0.881, whereas the subscales ranged between 0.69 and 0.87, representing a good internal consistency [38].

## Discussion

Both the global burden and incidence of thyroid cancer have been increasing in recent years with significant regional and gender-specific variations [40]. In Turkey, the age-standardised incidence rates of thyroid cancer were 22.1 per 100 000 women and 6.3 per 100 000 men in 2018 [41]. Previous studies on the long term impacts of ^131^I therapy reported that some socio-demographic and clinical factors affect patients’ health QoL negatively [12, 16, 17]. To our knowledge, this is the first study to evaluate OHRQoL and the factors affecting OHRQoL in patients receiving ^131^I therapy in the long term period. Compared to control patients, there was no significant difference in the total OHRQoL scores between groups, but patients receiving ^131^I therapy had worse periodontal parameters, and lower tooth brushing frequency. In a before after study of Köse et al. [42] with Turkish tyroid cancer patients receiving ^131^I therapy, patients’ periodontal parameter such as PI and BOP values found to be statistically higher than controls in short term period. Similarly, in current study, PI, GI, BOP, CAL, and PD values were found to be significantly lower in the control group compared to the ^131^I therapy group in the unadjusted bivariate analysis. Age adjusted analysis revealed significant differences only in GI and BOP between groups. Increasing scores of BOP and GI showed higher degree of gingival inflammation [43]. This may explained by a fact that the reducing salivary flow rate found in the ^131^I therapy group may reduce the cleaning and antibacterial activity of saliva and increase the accumulation of dental plaque.

In the unadjusted analysis, the DMFS value was found to be statistically lower in healthy controls than patients receiving ^131^I therapy, but no significant difference was found between groups in the DMFS value after adjusting for age. In various studies, similar to our study, an increase in the rate of xerostomia and dental caries was observed in individuals receiving ^131^I therapy [3, 44–46]. We found the salivary hyposecretion and lower tooth brushing frequency in patients receiving ^131^I therapy. This may lead to higher caries severity and periodontal diseases in these patients as a risk factor.

Although many studies evaluated the short-term side effects of ^131^I, small number of studies studied long-term side effects [3, 4, 44–48]. We found lower unstimulated and stimulated salivary flow rates in cancer patients compared to the control patients in both adjusted and unadjusted analyses, which was similar to the findings reported in previous studies on long-term salivary effects of ^131^I therapy [3, 4, 44–49]. Therefore, the results of this study support the necessity of a long-term follow-up in these patients [48].

In contrast, Ish-Shalom et al. [47]. reported that unstimulated salivary flow did not change in patients with ^131^I therapy. They reported that this may be explained by the fact that at rest, salivary secretion comes from the submandibular gland rather than the parotid gland and the submandibular gland is more resistant to radiation damage due to mucoid secretion [47].

A prospective cohort study with follow-up for up to 3 years reported that the rate of subjective xerostomia severity after ^131^I therapy was reduced in the third year of follow-up [49]. In a multicenter before-after prospective study, a decrease in unstimulated and stimulated salivary flow rates and an increase in the subjective feeling of xerostomia were found in the 5 months after treatment [50]. In contrast to the normative assessment, there was no difference between groups in the assessment of the subjective severity of xerostomia in this study. This finding shows that although cancer patients have poor salivary flow rate parameters, they do not perceive it. Further research is needed to assess the agreement between subjective and normative xerostomia severity and the wide range factors affecting this agreement.

In current study, the total OHIP-14 value was lower in the control group than in the ^131^I therapy group and there was no statistically significant difference between the groups. After adjusting for age, higher scores in the physical pain domain were found in the case group then control group. We found that patients with lower stimulated and unstimulated salivary flow rates had more psychological discomfort whereas patients with higher subjective xerostomia severity perceived worse OHRQoL, higher physical pain and psychological disability. This can be explained by the fact that patients experience some psychological and physical problems due to increased dental caries, periodontal problems, tooth loss, bad breath, and limiting oral functioning as a result of hyposalivation. One of the striking results of our study is that patients perceived better their OHRQoL and its domains of physical pain and psychological disability as the number of repeated ^131^I treatments increased. Ramim et al. [16] reported that patients experienced an improvement on their health related QoL (HRQoL) and symptoms reduction post-therapy. This may be related to patients’ psychological factors, the perception of hyposalivation and oral disease. It has been reported that hyposalivation is a subjective finding and patients may not feel it even when they have low saliva and that xerostomia may not always be associated with an actual reduction in saliva quality or quantity [51]. Some psychological problems such as stress, anxiety and depression may lead to xerostomia because of reducing unstimulated salivary flow rate [52]. Furthermore, the perception of dry mouth or xerostomia is considered a risk factor for dental and periodontal diseases, and the subjective feeling of dry mouth is one of the most important conditions that undermine patients’ quality of life [51]. Hyposalivation is generally observed as an early-term side effect after ^131^I therapy and it returns to its baseline value in the long term, and patients get used to this situation over time, even if there is hyposalivation. In addition, we found that healthy controls who had worse periodontal parameters perceived better OHRQoL, these control patients felt their QoL as good despite having periodontal disease. However, considering the limited salivary hypofunction observed in the experimental group, it is conceivable that salivary dysfunction due to radioiodine therapy has a minimal effect and that external factors may have a more significant impact on OHRQoL outcomes.

A small number of studies showed that Turkish thyroid cancer survivors had impaired the HRQoL [13, 15]. In the line with the results of the current study, Akyıldız et al. [15] reported that the low ability of Turkish patients to understand the disease affects their disease perception and QoL. The OHRQoL is a multidimensional construct which is affected by many factors as cultural values, attitudes, behaviors, and expectations throughout life [53, 54]. The patients included in this study had inadequate oral health behaviours. No differences were found between groups in terms of dental visiting pattern, self perceived periodontal health and treatment need but only diffence in the frequency of tootbrushing was observed between groups. Patients receiving ^131^I therapy reported less frequently toothbrushing than control patients. This finding may explain the higher periodontal paramaters observed in cancer patients. Most of them were problem-oriented attenders and they adapted to clinical changes by lowering their expectations throughout life.

Within common risk approach, oral diseases share the same risk factors with non-communicable chronic diseases. Therefore, oral health should be integrated into general health programs [55]. As risk factors, observing salivary hypofunction, having heavy dental plaque, worse oral health behaviours, limited oral health awareness in cancer patients highlights the need to include the oral helth professionals into a team-based care for cancer survivors [3, 6, 15, 53, 54].

The patients should take dental check-up and receive appropriate curative, preventive and rehabilitative treatment before and post ^131^I therapy. Dentists play an important role in the detection, monitoring and treatment of both the oral diseases and symptoms. In the line previous suggestions [19, 56], oral health professionals may assess unmet needs of patients and perform educational efforts to meet oral health educational needs.

The main strength of the study is the use of a patient-reported outcome measure to assess the impacts of the ^131^I therapy on patients’s OHRQoL. Internal consistency of OHIP-14 was found to be acceptable. Large oral health related outcomes including periodontal status, caries level, and oral health behaviours were evaluated together with subjective and objective salivary parameters and clinical records regarding the ^131^I therapy. The null hypothesis was rejected, since we found significant differences in salivary paramaters, oral health status and OHRQoL between groups.

However, the present study has some limitations. Firstly, the unmatched case-control study was conducted in a clinic of public dental faculty, with small sample size. The results are thus not generalizable. Study sample size was calculated based on the difference in the xerostomia proportions of 0.39 between control and study group [3, 20]. The post hoc power analyses showed that the achieved power is 86.7% for BOP, 75,3% for GI, and over 0.90 for stimulated and unstimulated salivary flow rates in the sample analyzed, considering an alpha of 0.05. Secondly, study sample consisted of female patients without systemic diseases. Due to higher age-standardised incidence rates of thyroid cancer in Turkish female [41], this study performed in a sample of female patients. Having systematic diseases applied as an exclusion criterion because it may be accepted a risk factor for both periodontal diseases and tyroid cancer. New multicenter matched case-control studies are warranted to control for confounding variables regarding basic socio-demographic variables and systemic diseases. In this study, we used the logictic regression analysis to control age covariate. Thirdly, longitudinal are recommended to investigate the cause-and-effect relationships. Fourthly, psychological factors, general and disease specific QoL were not evaluated in this study. Administration of generic and disease-specific validated questionnaires in future studies may provide valuable compherensive informations for health professionals when planning a team based cancer care [56]. Additionally, considering our inclusion criteria, since the sample size constituted a very small group, it was not possible to match age and individual tooth brushing habits, which are among the factors affecting periodontal health, between the groups. At the same time, by assessing the oral health status of patients before and after ^131^I therapy, possible complications of ^131^I therapy could be more ideally identified. In addition, matched case-control studies can be planned by increasing the sample size and selecting patients who are similar in terms of individual factors such as oral hygiene habits and age.

Finally, duration time after the ^131^I therapy and total ^131^I dose were found not correlated with the OHRQoL in this study. The multicenter prospective studies are warranted to compare the clinical symptoms by taking into account the factors influencing the formation of chronic sialoadenitis, the severity of the inflammatory process and patients’ QoL [12, 16, 17, 53, 54].

## Conclusion

The results of this study revealed that periodontal oral health status, tooth brushing frequency and OHRQoL of patients receiving ^131^I therapy were adversely affected compared with healthy control patients. Due to xerostomia, patients reported the worst OHRQoL in the domains of physical pain, psychological discomfort and disability. The findings of this study may provide a valuable insight on the oral health problems and needs of target group when planning a a team-based care.

## Data Availability

The data that support the findings of this study are not openly available due to reasons of sensitivity and are available from the corresponding author upon reasonable request.
